# Supplementary figures and images for: Maternal counseling for preterm deliveries, assessing an effective method of counseling: A randomized trial
Source: PLoS One. 2025 Apr 9;20(4):e0294168. doi: 10.1371/journal.pone.0294168 (PMC11981203; doi:10.1371/journal.pone.0294168)

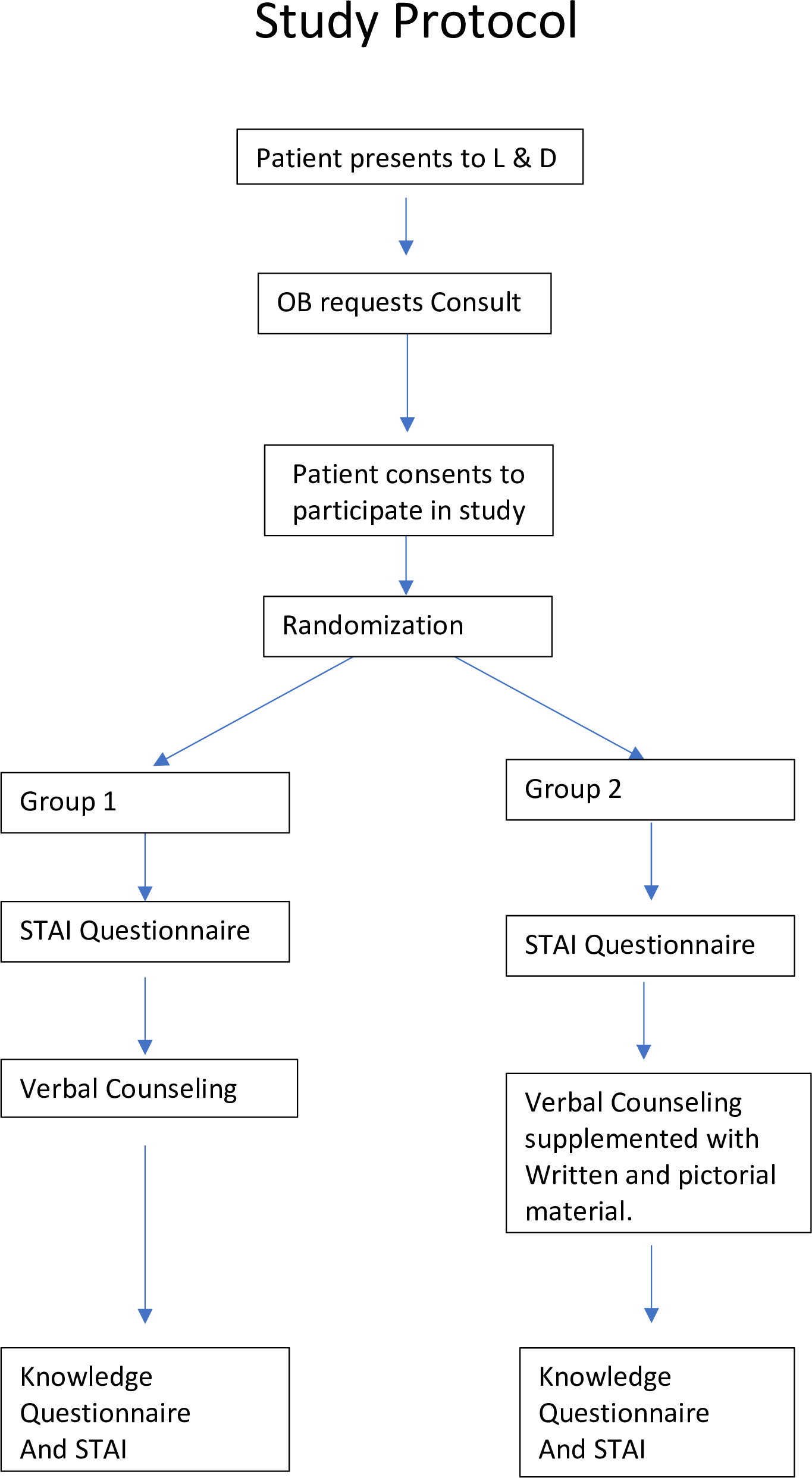

Supplement: S1 Protocol — Study Protocol. (TIF) [file pone.0294168.s001.tif]

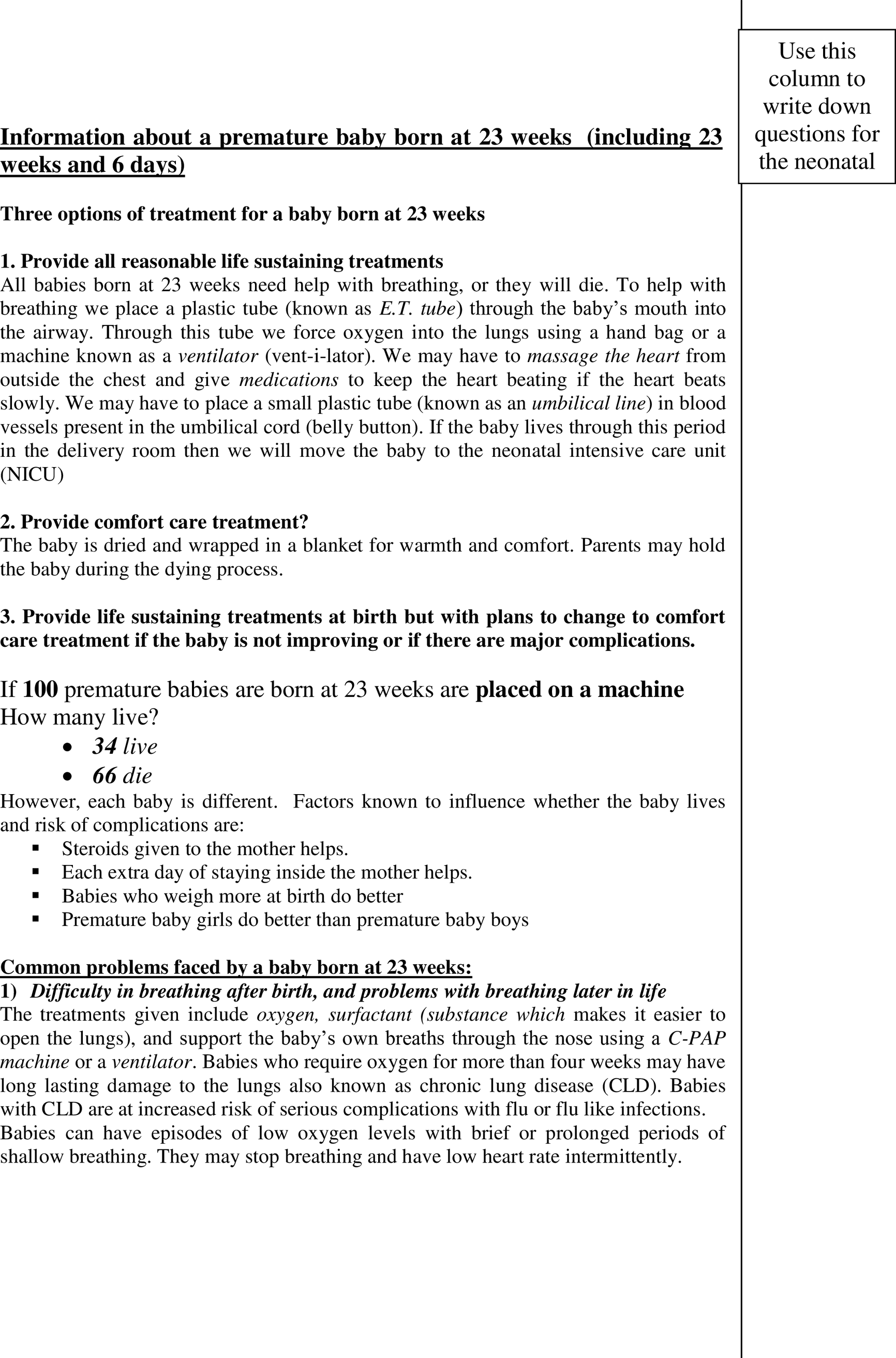

Supplement: S1 Appendix — Sample counseling material. (ZIP) [file pone.0294168.s002.zip › PACE Corrected/Information about a premature baby born at 23 weeks..tif]

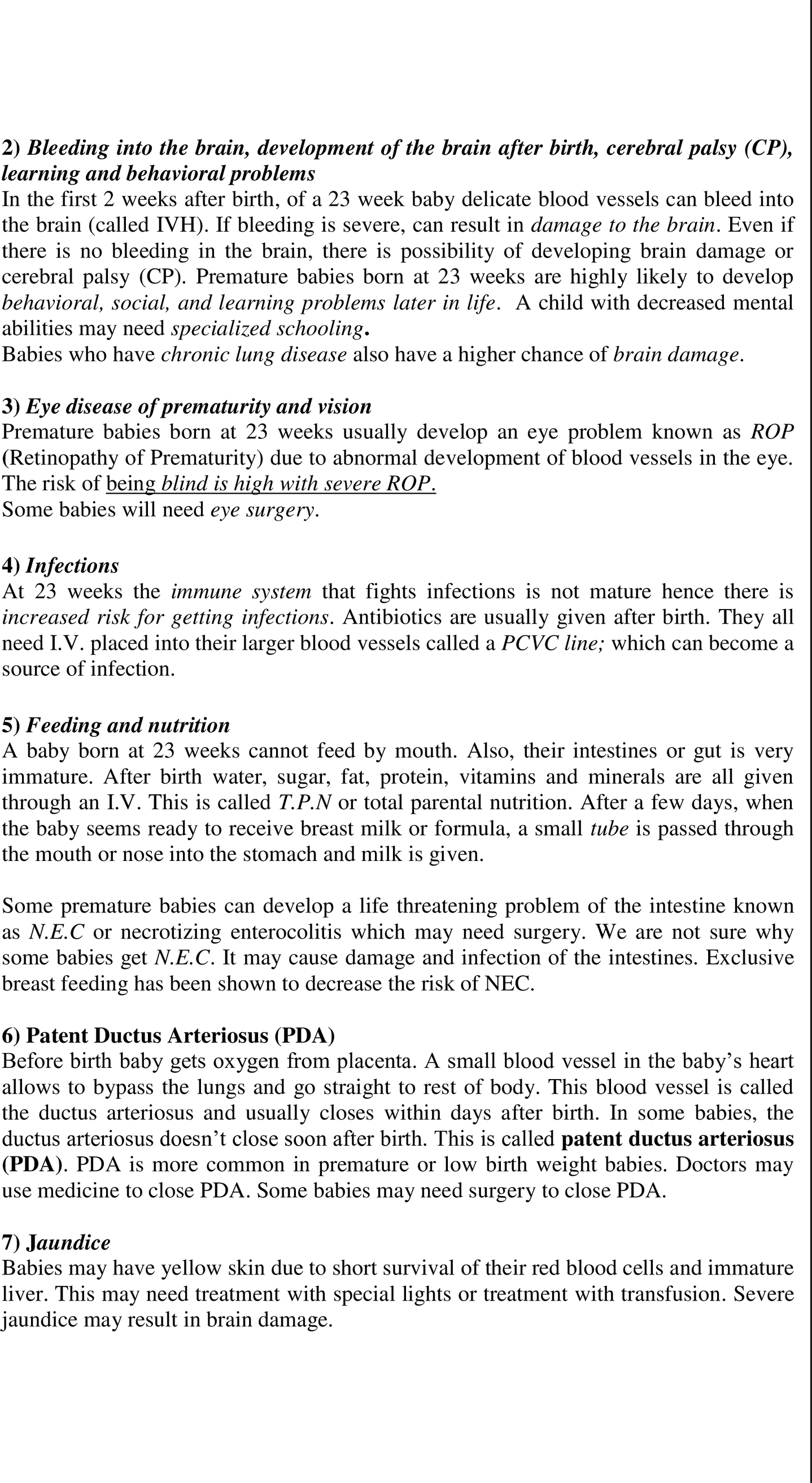

Supplement: S1 Appendix — Sample counseling material. (ZIP) [file pone.0294168.s002.zip › PACE Corrected/Information about a premature baby born at 23 weeks..tif]

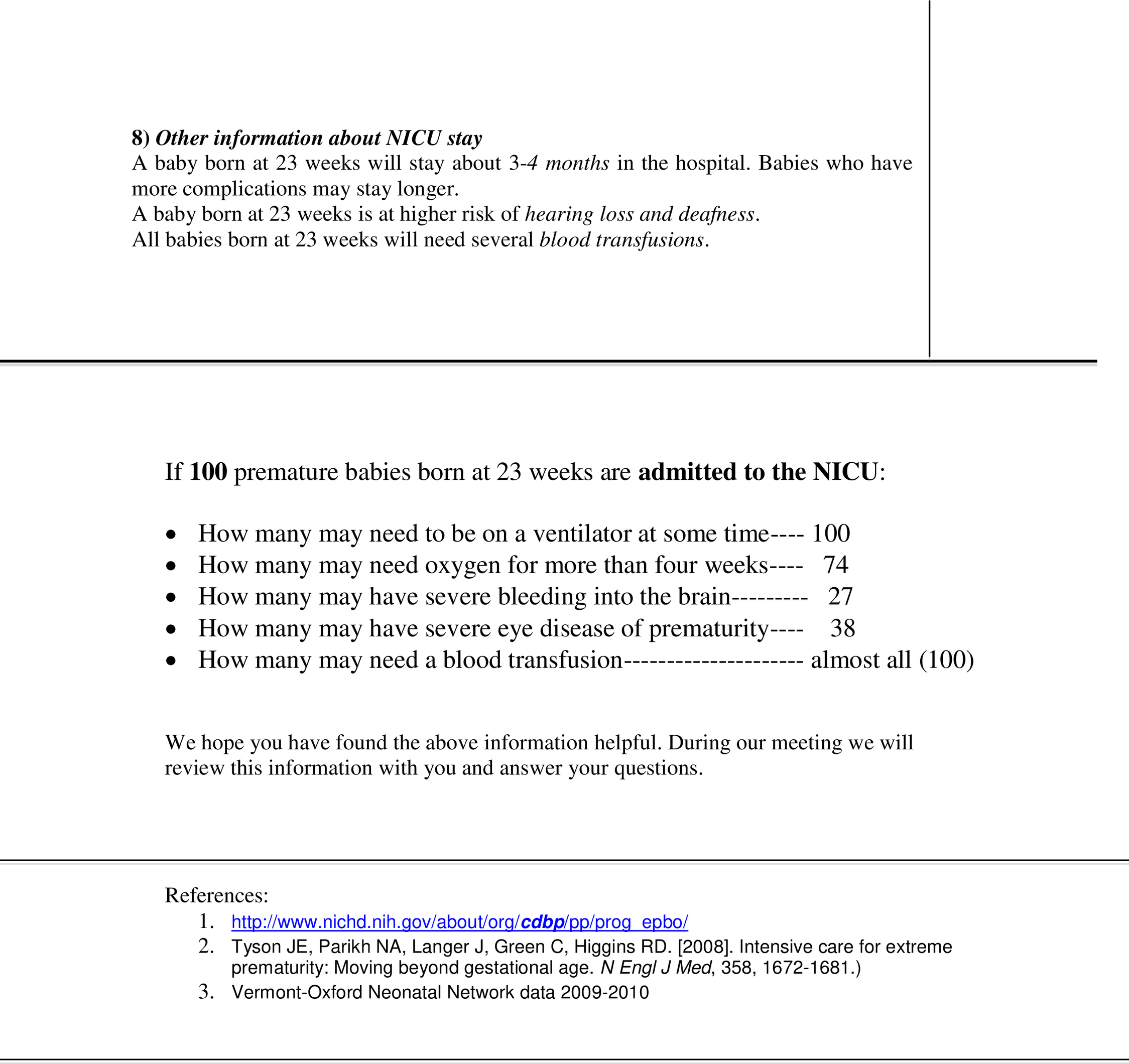

Supplement: S1 Appendix — Sample counseling material. (ZIP) [file pone.0294168.s002.zip › PACE Corrected/Information about a premature baby born at 23 weeks..tif]

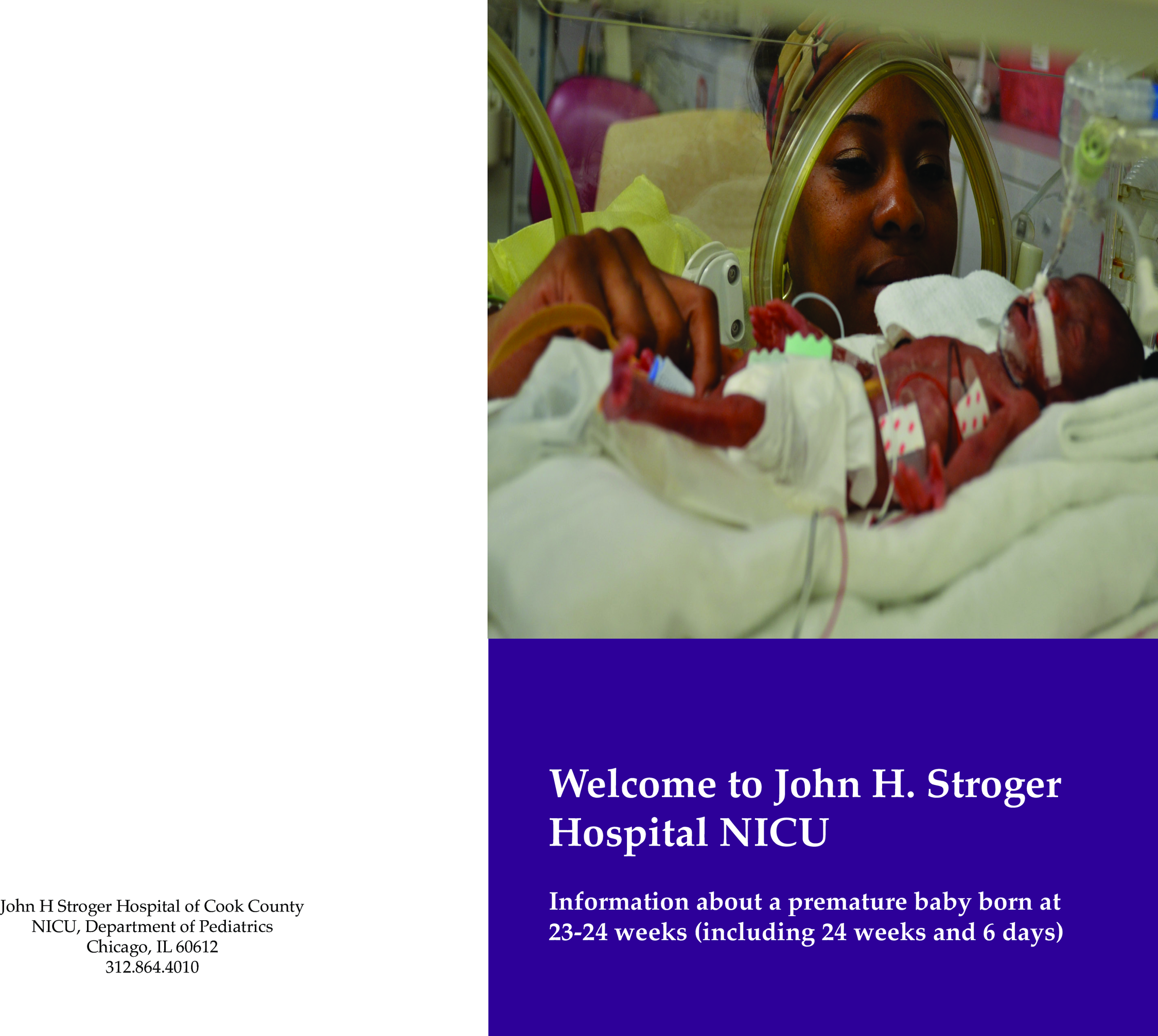

Supplement: S1 Fig.pdf — Sample picture booklet. (ZIP) [file pone.0294168.s003.zip › PACE Corrected/23-24 booklet.tif]

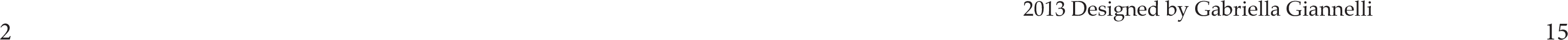

Supplement: S1 Fig.pdf — Sample picture booklet. (ZIP) [file pone.0294168.s003.zip › PACE Corrected/23-24 booklet.tif]

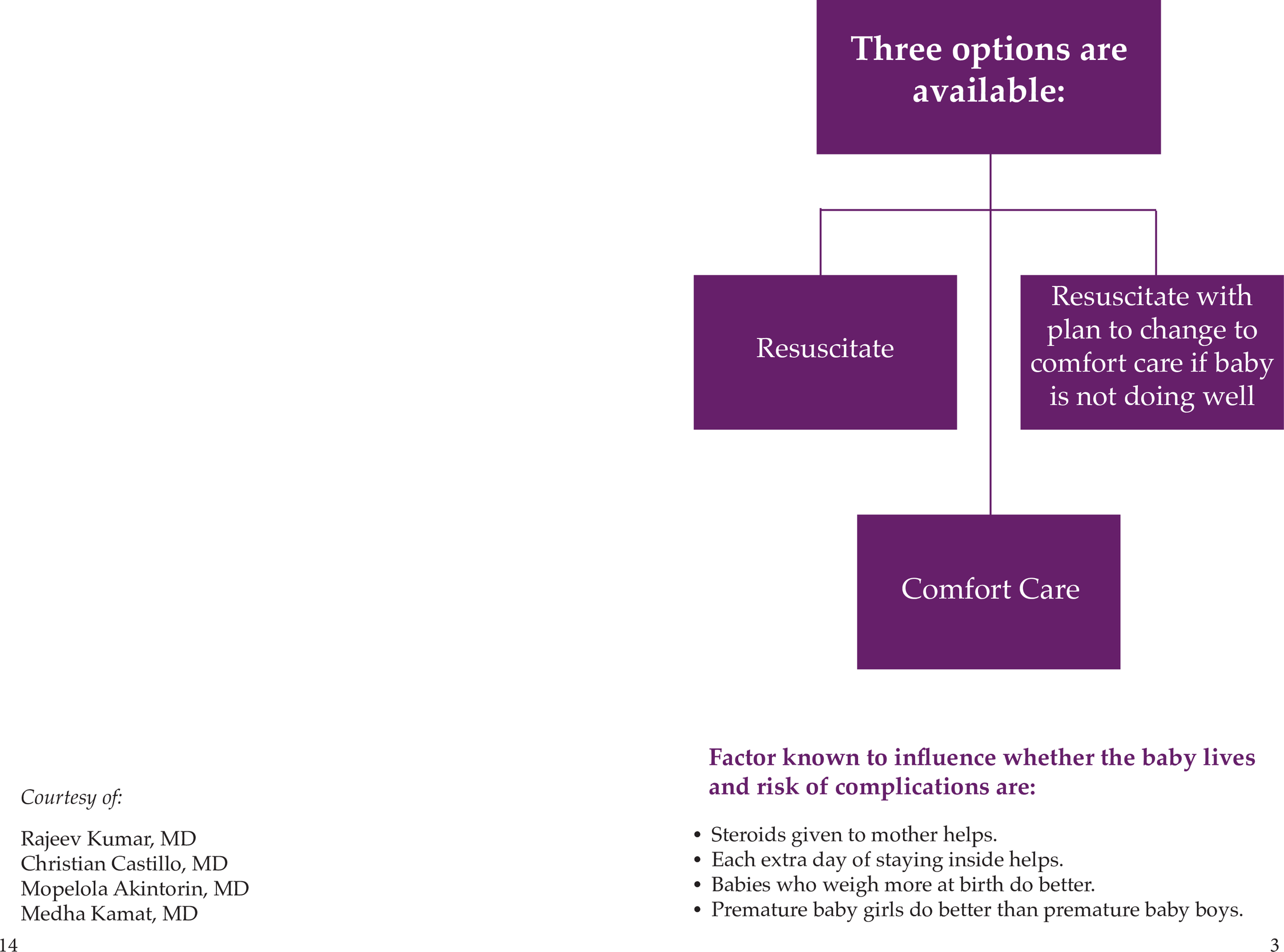

Supplement: S1 Fig.pdf — Sample picture booklet. (ZIP) [file pone.0294168.s003.zip › PACE Corrected/23-24 booklet.tif]

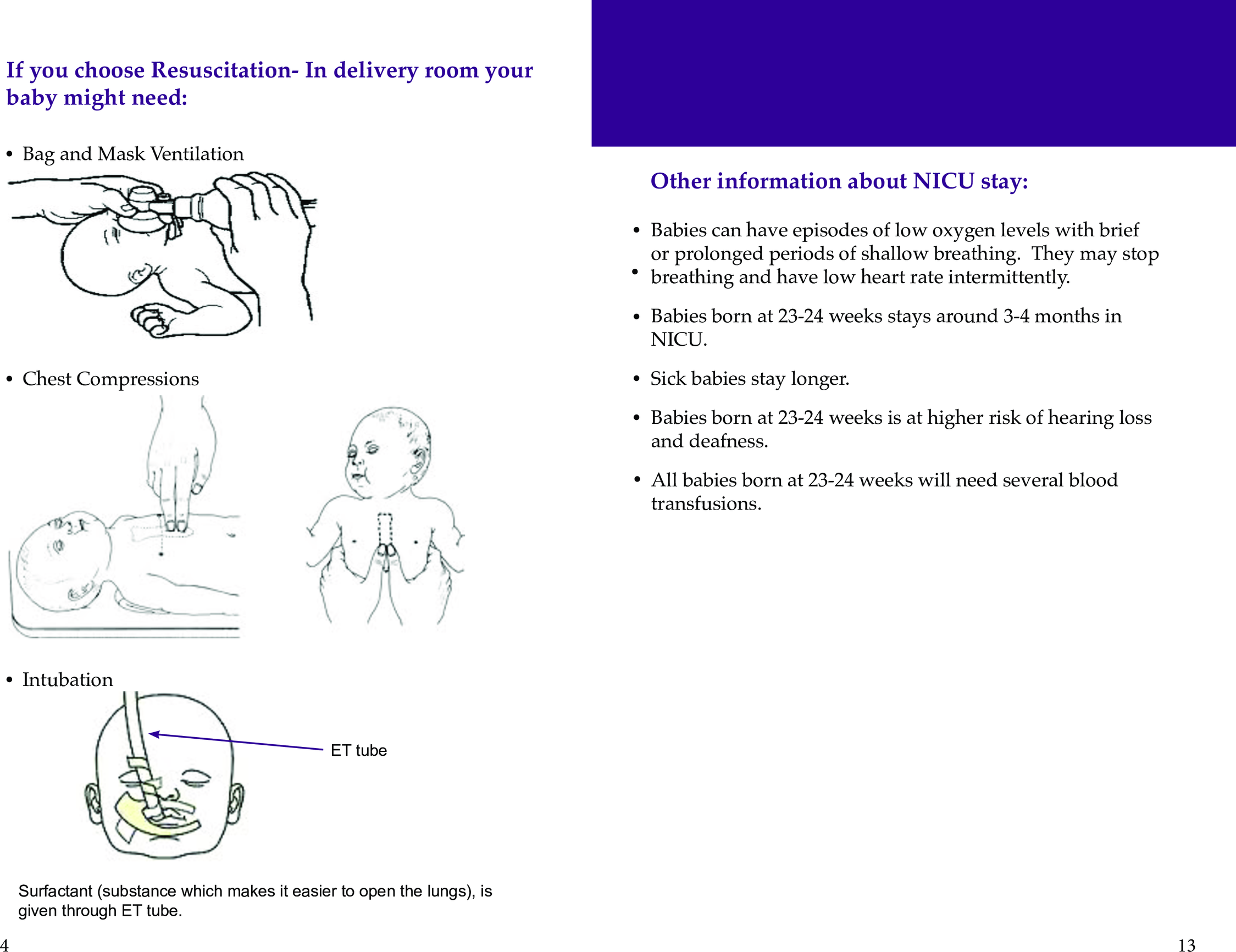

Supplement: S1 Fig.pdf — Sample picture booklet. (ZIP) [file pone.0294168.s003.zip › PACE Corrected/23-24 booklet.tif]

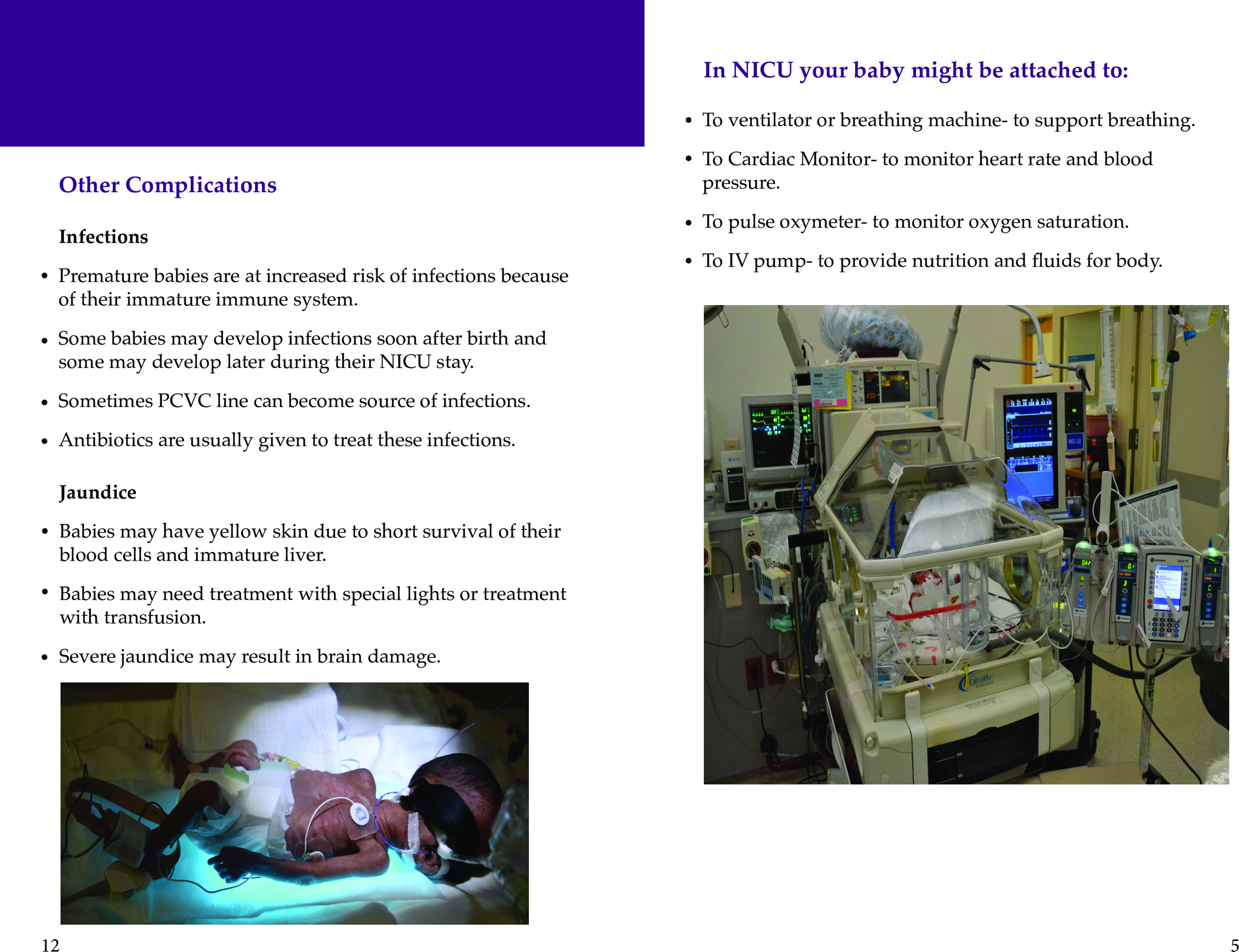

Supplement: S1 Fig.pdf — Sample picture booklet. (ZIP) [file pone.0294168.s003.zip › PACE Corrected/23-24 booklet.tif]

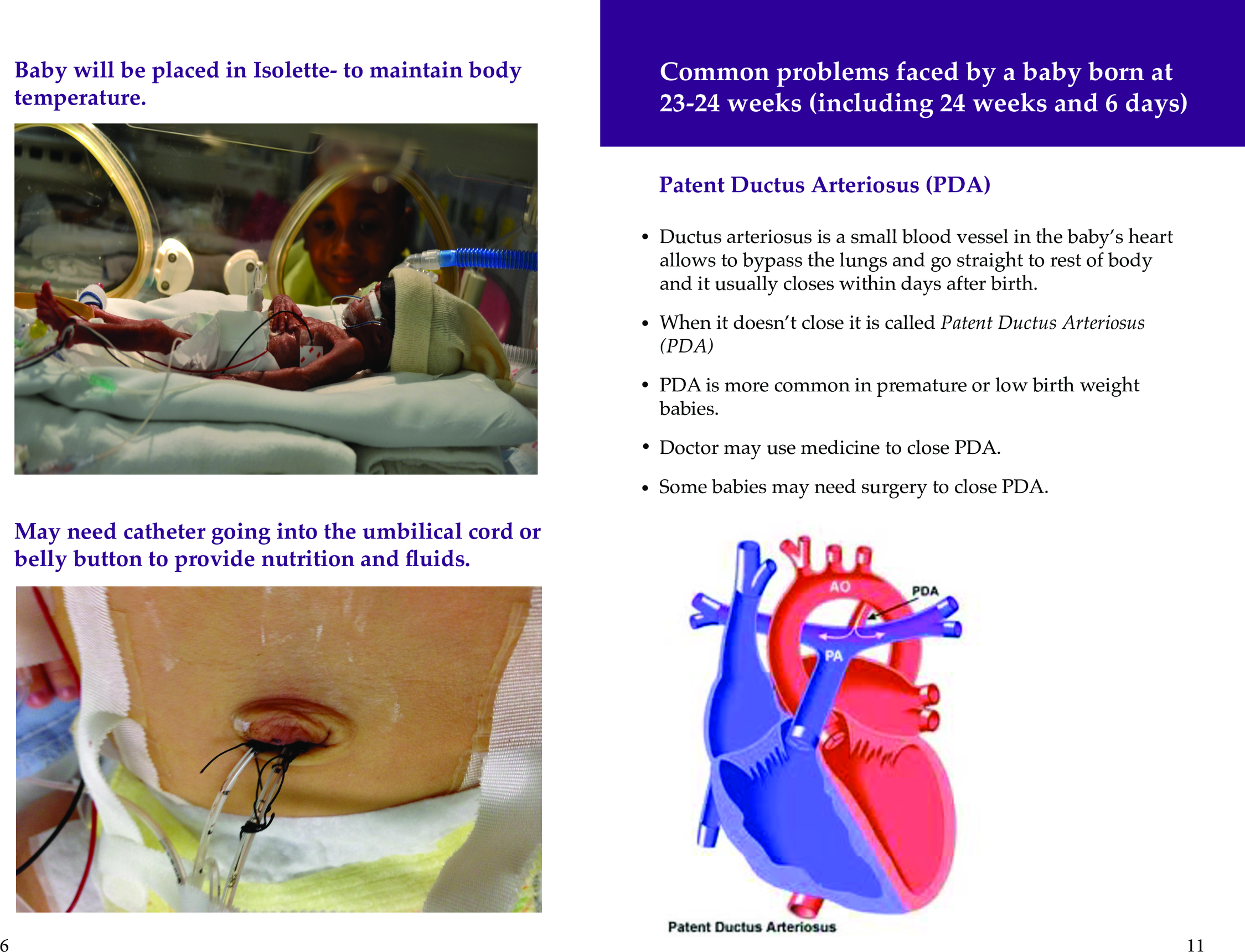

Supplement: S1 Fig.pdf — Sample picture booklet. (ZIP) [file pone.0294168.s003.zip › PACE Corrected/23-24 booklet.tif]

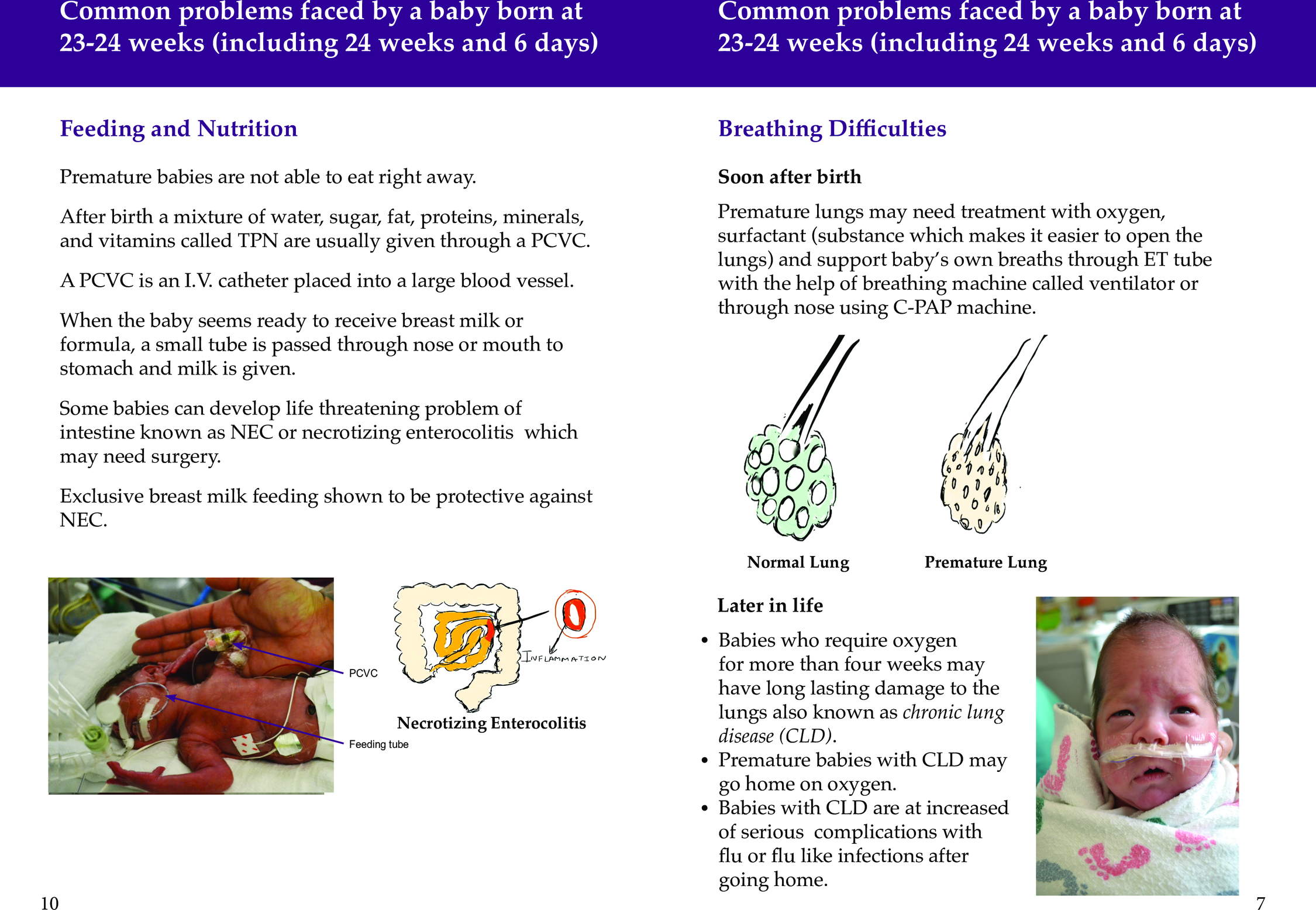

Supplement: S1 Fig.pdf — Sample picture booklet. (ZIP) [file pone.0294168.s003.zip › PACE Corrected/23-24 booklet.tif]

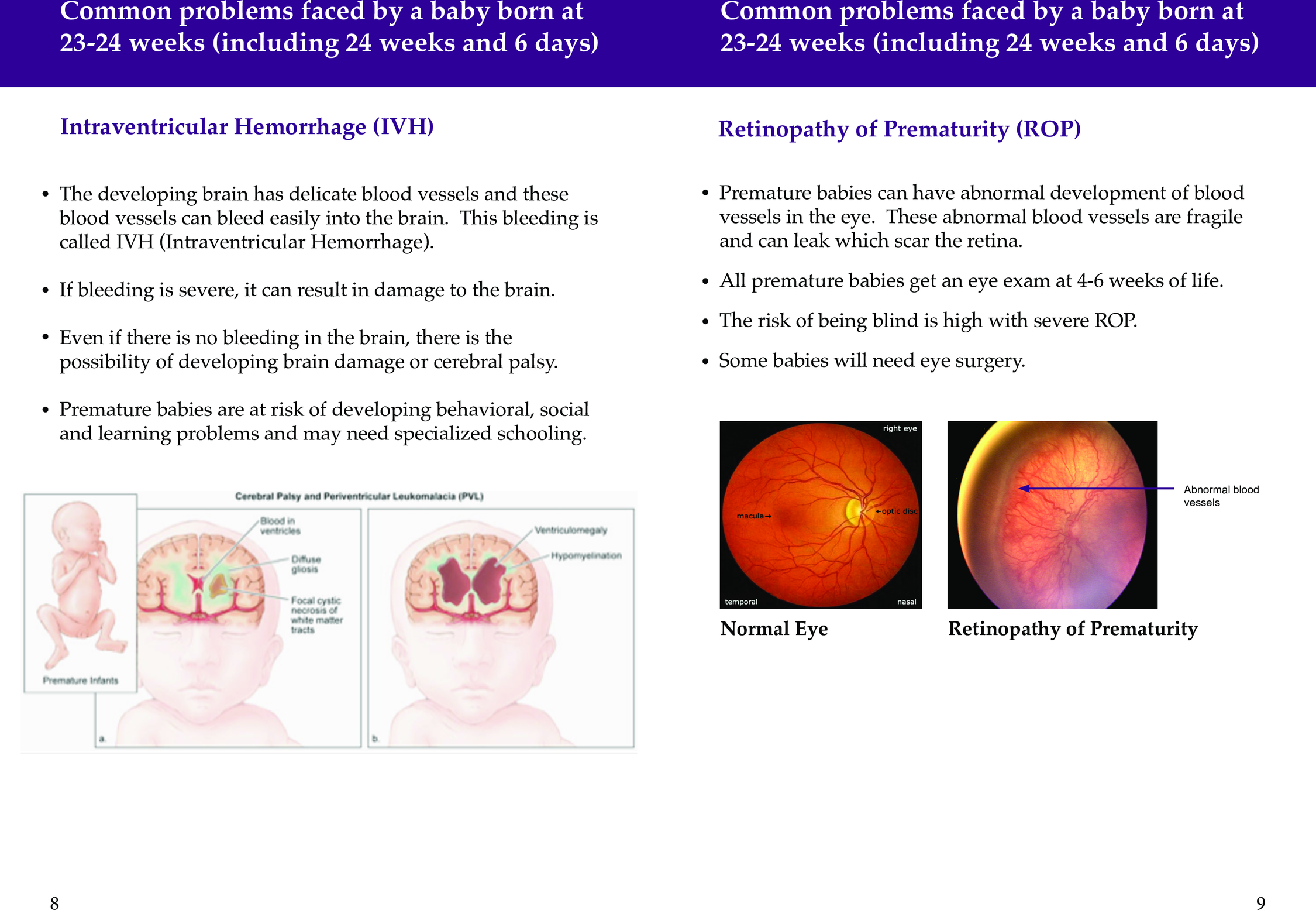

Supplement: S1 Fig.pdf — Sample picture booklet. (ZIP) [file pone.0294168.s003.zip › PACE Corrected/23-24 booklet.tif]

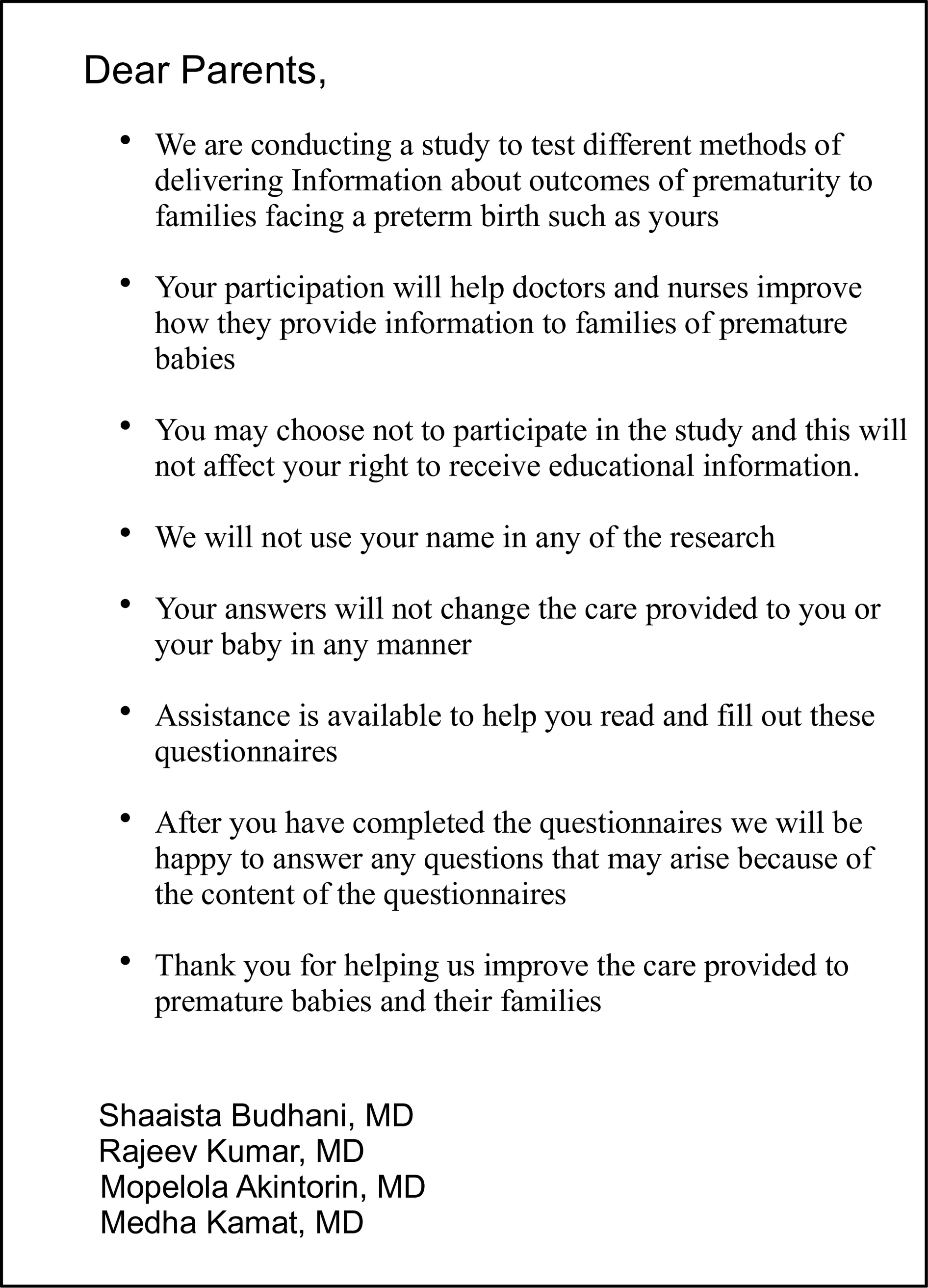

Supplement: S2 Appendix — Sample questionnaire. (ZIP) [file pone.0294168.s004.zip › PACE Corrected/Questionnaire.tif]

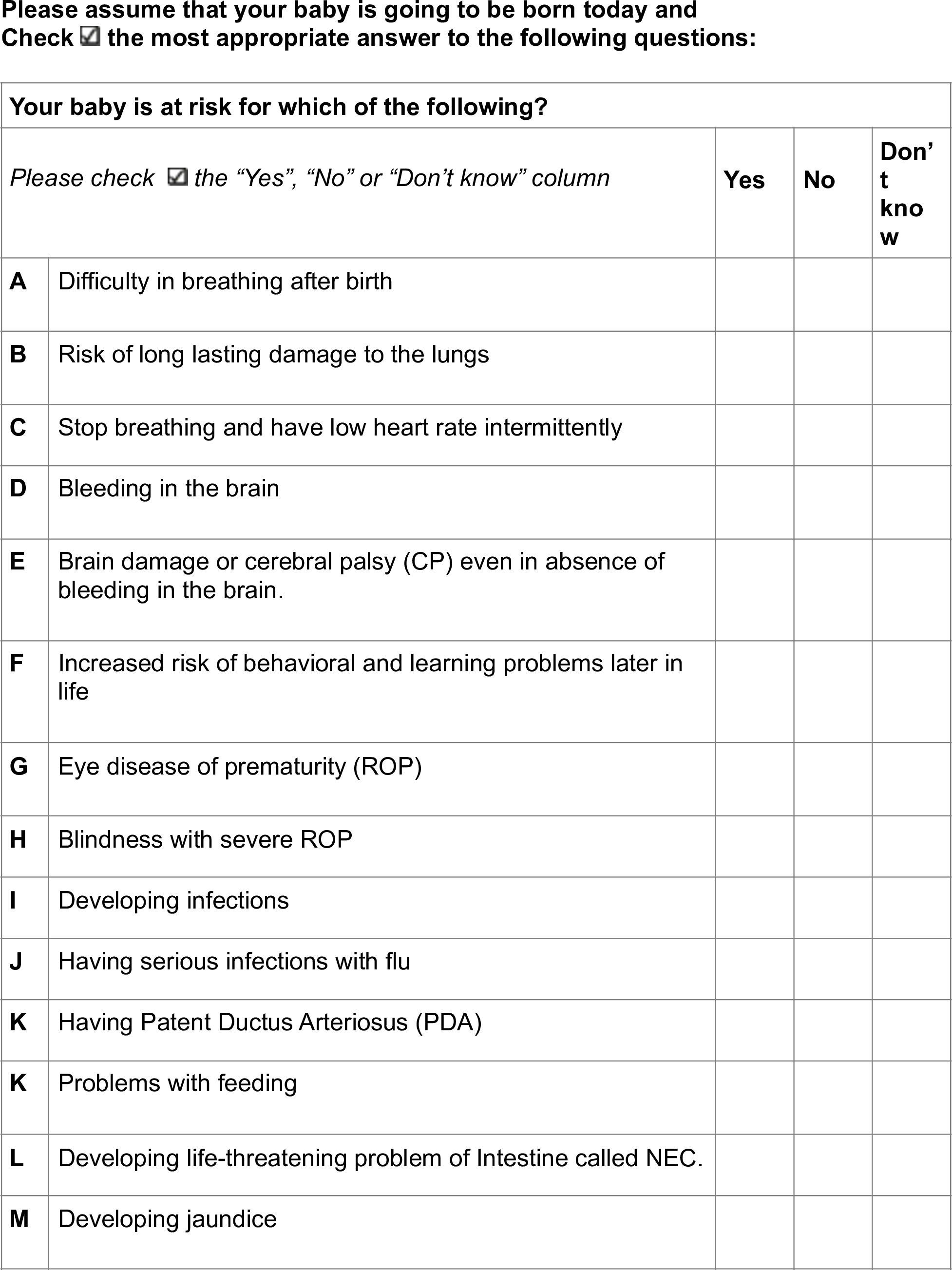

Supplement: S2 Appendix — Sample questionnaire. (ZIP) [file pone.0294168.s004.zip › PACE Corrected/Questionnaire.tif]

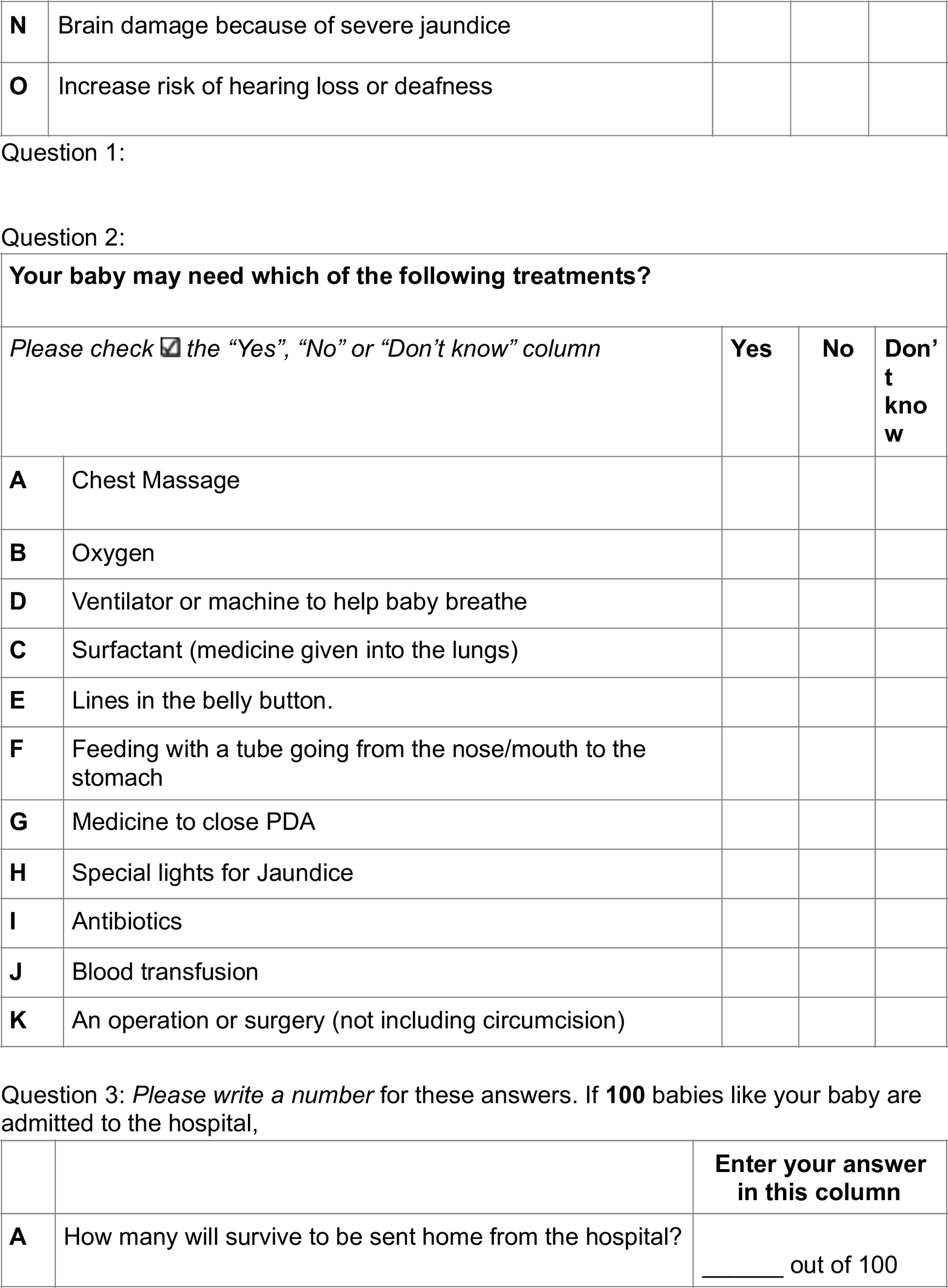

Supplement: S2 Appendix — Sample questionnaire. (ZIP) [file pone.0294168.s004.zip › PACE Corrected/Questionnaire.tif]

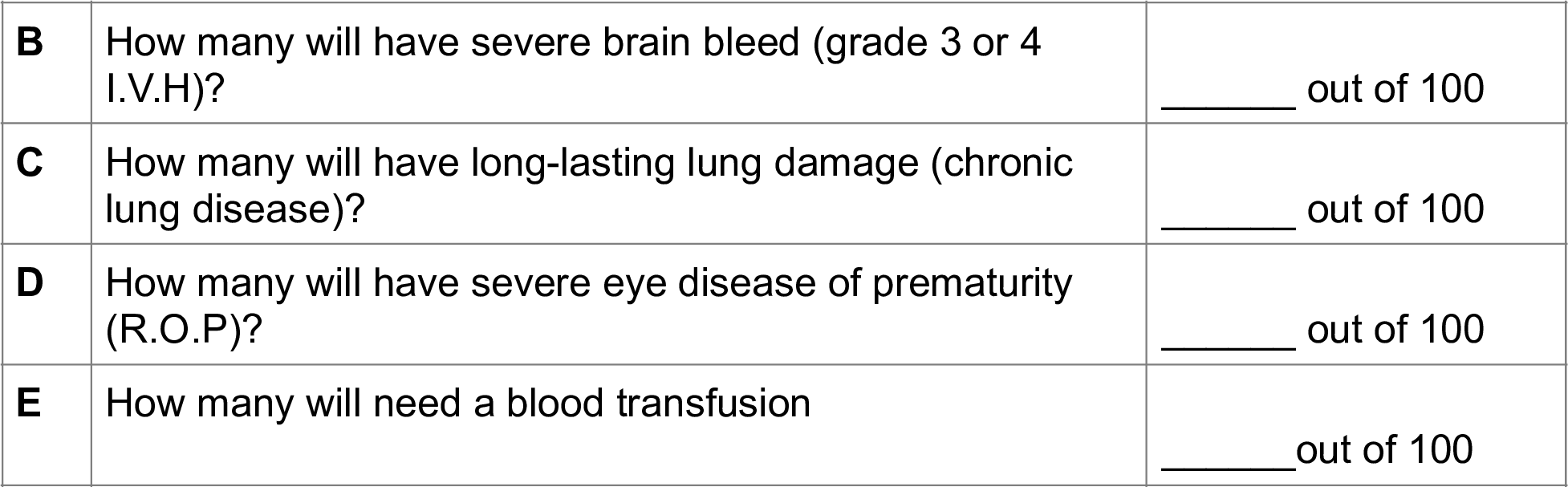

Supplement: S2 Appendix — Sample questionnaire. (ZIP) [file pone.0294168.s004.zip › PACE Corrected/Questionnaire.tif]
