# Supplementary material for: Maternal counseling for preterm deliveries, assessing an effective method of counseling: A randomized trial
Source: PLoS One. 2025 Apr 9;20(4):e0294168. doi: 10.1371/journal.pone.0294168 (PMC11981203; doi:10.1371/journal.pone.0294168)
Supplement: Attachment — Submitted filename: Rebuttal letter 2-28-2024.docx [file pone.0294168.s007.docx]

Friday, March 1st, 2024

To the Editor

PLOS ONE

Dear Editor,

**Ref: MATERNAL COUNSELING FOR PRETERM DELIVERIES, ASSESSING AN EFFECTIVE METHOD OF COUNSELING: A RANDOMIZED TRIAL.**

Dear Editors and Reviewers,

Thank you for your time and consideration in reviewing our manuscript. We appreciate your feedback. All comments have been addressed. Please see below for our responses.

If questions arise, please do not hesitate to email rkumar3@cookcountyhhs.org

Truly

Rajeev Kumar

Major Changes

Test the interaction of group by time rather than repeatedly testing differences in time and by group. If the interaction effect is significant, provide an interpretation of the results, but do not test main effects because the tests for main effects are uninteresting in light of significant interactions. If interaction effects are non-significant, drop the interaction effects from the model and test the main effects. Determining which results to present when testing interactions is often a multi-step process.

Response: We have read this comment several times and we do not understand what the ask is

Study Design section: Indicate if block randomization was used. If so, state the block size.

Response: We did not use block randomization

State the alpha level and statistical testing method used in sample size calculation.

Response: alpha level was 0.05 study power of 0.80 to detect group differences of 0.25

Qualitative variables are typically expressed as frequencies and percentages.

Response: Corrected now reads “ Qualitative variables were expressed as frequencies and percentages

The independent t-test is written as “t-test”.

Response: Corrected now reads t-test

The complete name of the Mann-Whitney test is the Mann-Whitney U test and it is used to compare differences in central tendencies rather than means.

Response: Corrected now reads Mann-Whitney U test was used to compare differences in central tendencies

The following sentence is unclear: “The p tests for the Shapiro-Wilk test for the continuous variables were p = 0.07 for Age; p = 0.00 for hours, gravida, and gestational age; p = 0.01 and 0.03 for pre and Post STAI scores meaning that these variables were not normally distributed.”

Response: statement removed

P-values never equal zero; express small p-values as < 0.001.

Response: Corrected small p values now reported as < 0.001

The Wilcoxon Signed Rank test is a nonparametric test which compares central tendencies between two groups.

Response: Noted and corrected

Add numeric p-values to Table 1.

Response: Done

Cite the statistical software used for the analysis.

Response: Stata 17

Thoroughly proofread the manuscript. Provide as much clarity as possible. A few noted grammatical mistakes follow.

Response: Done

Study Design section: Be consistent with formats for the months of January and September.

Response: Corrected

- A few sentences are missing a period at the end.

Response: Manuscript revised and corrected

To assist in the review process, add line numbering to the document.

Response: line number added
