# Supplementary material for: Maternal counseling for preterm deliveries, assessing an effective method of counseling: A randomized trial
Source: PLoS One. 2025 Apr 9;20(4):e0294168. doi: 10.1371/journal.pone.0294168 (PMC11981203; doi:10.1371/journal.pone.0294168)
Supplement: Attachment — Submitted filename: Rebuttal letter.docx [file pone.0294168.s006.docx]

**MATERNAL COUNSELING FOR PRETERM DELIVERIES, ASSESSING AN EFFECTIVE METHOD OF COUNSELING: A RANDOMIZED TRIAL.**

Dear Editors and Reviewers,

Thank you for your time and consideration in reviewing our manuscript. We appreciate your feedback. All comments have been addressed. Please see below for our responses.

Comments:

1. Please ensure that your manuscript meets PLOS ONE's style requirements, including those for file naming. The PLOS ONE style templates can be found at

<https://journals.plos.org/plosone/s/file?id=wjVg/PLOSOne_formatting_sample_main_body.pdf> and

[https://journals.plos.org/plosone/s/file?id=ba62/PLOSOne_formatting_sample_title_authors_affiliations.pdf](https://nam12.safelinks.protection.outlook.com/?url=https%3A%2F%2Fjournals.plos.org%2Fplosone%2Fs%2Ffile%3Fid%3Dba62%2FPLOSOne_formatting_sample_title_authors_affiliations.pdf&data=05%7C01%7Crkumar3%40cookcountyhhs.org%7Cdeccdb1289504bc8255708db939cb766%7C3b922295e886417faaa84e4c4f069d82%7C0%7C0%7C638266074504248787%7CUnknown%7CTWFpbGZsb3d8eyJWIjoiMC4wLjAwMDAiLCJQIjoiV2luMzIiLCJBTiI6Ik1haWwiLCJXVCI6Mn0%3D%7C3000%7C%7C%7C&sdata=xC4QuIGQqyKyl1zc7aefgG7k7r%2Fg4gZcNGWUVdZP2M8%3D&reserved=0).

Response: Manuscript has been updated to meet PLOS ONE’s style as required.

2. In the ethics statement in the Methods, you have specified that verbal consent was obtained. Please provide additional details regarding how this consent was documented and witnessed, and state whether this was approved by the IRB

Response: Verbal consent explanation has been updated in the manuscript.

3. We noticed you have some minor occurrence of overlapping text with the following previous publication(s), which needs to be addressed:

- <https://doi.org/10.1016/j.jpeds.2016.08.006>

- <https://doi.org/10.1542/peds.2015-2336>

- <http://weirdthings.com/t8oxgd0/interpretation-of-wilcoxon-signed-rank-test>

Response: Plagiarism has been addressed.

In your revision ensure you cite all your sources (including your own works), and quote or rephrase any duplicated text outside the methods section. Further consideration is dependent on these concerns being addressed.

Response: Duplicated text has been edited all the work has been cited (9,10).

4. Please include your ethics statement in the Methods section of your manuscript. In the Methods section of your revised manuscript, please include the full name of the institutional review board or ethics committee that approved the protocol, the approval or permit number that was issued, and the date that approval was granted.

Response: Ethics statement has been added. IRB name has been added.

5. Please upload the trial protocol and the ethics documents you submitted as separate files.

Response: Protocol and Ethics documents have been submitted as separate files.

6. We note that you have stated that you will provide repository information for your data at acceptance. Should your manuscript be accepted for publication, we will hold it until you provide the relevant accession numbers or DOIs necessary to access your data. If you wish to make changes to your Data Availability statement, please describe these changes in your cover letter and we will update your Data Availability statement to reflect the information you provide.

Response: Data has been uploaded to Mendley.

Soyemi, Kenneth (2023), “MATERNAL COUNSELING FOR PRETERM DELIVERIES, ASSESSING AN EFFECTIVE METHOD OF COUNSELING: A RANDOMIZED TRIAL.”, Mendeley Data, V1, doi: 10.17632/ry4v5b3f2h.1

7. Please include a separate caption for each figure in your manuscript.

Response: Each figure has its own caption now. Figure has been moved to supplemental information.

8. Please upload a copy of Figure 2, to which you refer in your text on page 7. If the figure is no longer to be included as part of the submission please remove all reference to it within the text.

Response: Figure 2 reference has been removed.

9. Please include captions for your Supporting Information files at the end of your manuscript, and update any in-text citations to match accordingly. Please see our Supporting Information guidelines for more information: <http://journals.plos.org/plosone/s/supporting-information>.

Response: Captions for supporting documents have been added at the end of manuscript.

S1 Protocol: Study Protocol.

S1 Appendix: Sample counseling material

S1_Fig.pdf: Sample picture booklet

S2 Appendix: Sample questionnaire

Additional Editor Comments (if provided):

Abstract
Major comment: The results section within the abstract seems to focus on the secondary outcome. It does not present actual results for the primary outcome. Only a conclusion is presented saying there was no significant difference in recall rates between the two groups (which means the two counseling methods had similar effectiveness). Please present in a very concise manner the actual rates or measures of effect and/or p-values on which this conclusion is based. The results section should share the actual results on which the conclusion is based, as the abstract is stand-alone.

Response: Result section has been modified to reflect primary outcome.

Introduction
Minor comment: As you present the purpose of the study at the end of the introduction, please very briefly touch on specifically assessing “recall rates” for your primary outcome. That is the main element you evaluated.

Response: Introduction was modified to reflect “Recall rates” as primary outcome.
Minor comment: You have presented the purpose as a question. Please end with a question mark for punctuation.
Response: Punctuation has been added.
Methods
Minor comment: Please provide a reference for the validated knowledge questionnaire which you mention was used in a previously published study (reference to that previous study)
Minor comment: Please provide a reference for the Spanish translation of the same knowledge questionnaire which you mention you validated in a pilot study at your institution (reference to this validation pilot study for the Spanish version)
Response: Reference citation has been added for knowledge questionnaire on page. (6)

Response: Reference and citation added for Spanish questionnaire. (10)
Results
Major comment: You present secondary outcome (anxiety score) before the primary outcome, and in more detail. The primary outcome (recall rates) is only found as one sentence (more of a conclusion) in the last paragraph of the results section; there is no table or any number provided on the primary outcome. Yet as the primary outcome even your sample size calculation was based on detecting a knowledge difference between the two groups. Please swap around. Start with the primary outcome (recall rates) and provide adequate results on recall rates across the two groups for short term problems, long term problems, intervention, and incidence rates (provide numbers or even a table, and not just one concluding sentence in the last paragraph)

Response: Result section modified to reflect primary outcome and table (table 2) was added to reflect the results of primary with p values.

Minor comment: I am not sure what Figure 2 refers to. Please double check if there should be a reference to figure 2.

Response: Figure 2 refence was removed.


[Note: HTML markup is below. Please do not edit.]

Reviewers' comments:

Reviewer's Responses to Questions

**Comments to the Author**

1. Is the manuscript technically sound, and do the data support the conclusions?

The manuscript must describe a technically sound piece of scientific research with data that supports the conclusions. Experiments must have been conducted rigorously, with appropriate controls, replication, and sample sizes. The conclusions must be drawn appropriately based on the data presented.

Reviewer #1: Yes

2. Has the statistical analysis been performed appropriately and rigorously?

Reviewer #1: I Don't Know

3. Have the authors made all data underlying the findings in their manuscript fully available?

The [PLOS Data policy](https://nam12.safelinks.protection.outlook.com/?url=http%3A%2F%2Fwww.plosone.org%2Fstatic%2Fpolicies.action%23sharing&data=05%7C01%7Crkumar3%40cookcountyhhs.org%7Cdeccdb1289504bc8255708db939cb766%7C3b922295e886417faaa84e4c4f069d82%7C0%7C0%7C638266074504248787%7CUnknown%7CTWFpbGZsb3d8eyJWIjoiMC4wLjAwMDAiLCJQIjoiV2luMzIiLCJBTiI6Ik1haWwiLCJXVCI6Mn0%3D%7C3000%7C%7C%7C&sdata=Fg61iVG70jM3flw7dFwaxCo9kb7tRFqRkkiLx1CQJsA%3D&reserved=0) requires authors to make all data underlying the findings described in their manuscript fully available without restriction, with rare exception (please refer to the Data Availability Statement in the manuscript PDF file). The data should be provided as part of the manuscript or its supporting information, or deposited to a public repository. For example, in addition to summary statistics, the data points behind means, medians and variance measures should be available. If there are restrictions on publicly sharing data—e.g. participant privacy or use of data from a third party—those must be specified.

Reviewer #1: Yes

4. Is the manuscript presented in an intelligible fashion and written in standard English?

PLOS ONE does not copyedit accepted manuscripts, so the language in submitted articles must be clear, correct, and unambiguous. Any typographical or grammatical errors should be corrected at revision, so please note any specific errors here.

Reviewer #1: Yes

5. Review Comments to the Author

Please use the space provided to explain your answers to the questions above. You may also include additional comments for the author, including concerns about dual publication, research ethics, or publication ethics. (Please upload your review as an attachment if it exceeds 20,000 characters)

Reviewer #1: The paper reports on a small study which compares the effectiveness of verbal counselling versus verbal plus supplemental material for women threatened with preterm delivery. They found no significant differences. The study was reasonably well carried out but is not of much general interest to the broader medical/scientific community.

Response: Counseling of parents facing a threatened preterm delivery is a big challenge for neonatologists, as anxiety levels are very high. Parents are very emotional at this time, as there life is about to change and they could be numb to information provided. This study explored the methods of delivering vast information in concise and easily understandable way to a innercity population. This study adds to pool of existing studies and raises a point of exploring methods that deliver information in more effective way.

Other points.
There was no assessment of the literacy abilities of the individual in the two groups, and whether that affected their understanding of the written materials.

Response: Parents education level taken as proxy of their literacy abilities.

The STAI inventory (p6). is the short version of the STAI used based on the State rather than the Trait scale?

Response: STAI inventory (p6) has a correlation coefficient of 0.9 with full version and is used in situations where full form is inappropriate similar to our study population

Marteau TM, Bekker H. The development of a six-item short-form of the state scale of the Spielberger State-Trait Anxiety Inventory (STAI). Br J Clin Psychol. 1992 Sep;31(3):301-6. doi: 10.1111/j.2044-8260.1992.tb00997.x. Erratum in: Br J Clin Psychol. 2020 Jun;59(2):276. PMID: 1393159.

6. PLOS authors have the option to publish the peer review history of their article ([what does this mean?](https://nam12.safelinks.protection.outlook.com/?url=https%3A%2F%2Fjournals.plos.org%2Fplosone%2Fs%2Feditorial-and-peer-review-process%23loc-peer-review-history&data=05%7C01%7Crkumar3%40cookcountyhhs.org%7Cdeccdb1289504bc8255708db939cb766%7C3b922295e886417faaa84e4c4f069d82%7C0%7C0%7C638266074504248787%7CUnknown%7CTWFpbGZsb3d8eyJWIjoiMC4wLjAwMDAiLCJQIjoiV2luMzIiLCJBTiI6Ik1haWwiLCJXVCI6Mn0%3D%7C3000%7C%7C%7C&sdata=WhxUwLcDo2I7C59gdOlFM4nOfDIQyWzZQtyhral6dHE%3D&reserved=0)). If published, this will include your full peer review and any attached files.


If you choose “no”, your identity will remain anonymous but your review may still be made public.


**Do you want your identity to be public for this peer review?** For information about this choice, including consent withdrawal, please see our [Privacy Policy](https://nam12.safelinks.protection.outlook.com/?url=https%3A%2F%2Fwww.plos.org%2Fprivacy-policy&data=05%7C01%7Crkumar3%40cookcountyhhs.org%7Cdeccdb1289504bc8255708db939cb766%7C3b922295e886417faaa84e4c4f069d82%7C0%7C0%7C638266074504248787%7CUnknown%7CTWFpbGZsb3d8eyJWIjoiMC4wLjAwMDAiLCJQIjoiV2luMzIiLCJBTiI6Ik1haWwiLCJXVCI6Mn0%3D%7C3000%7C%7C%7C&sdata=LRp25aMaUvUpQmGk9M9n8XbvLTXhV68Fm2SZIxw2x9s%3D&reserved=0).

Reviewer #1: No

6. Muthusamy AD, Leuthner S, Gaebler-Uhing C, Hoffmann RG, Li SH, Basir MA. Supplemental written information improves prenatal counseling: a randomized trial. Pediatrics. 2012;129(5):e1269-74. Epub 20120409. doi: 10.1542/peds.2011-1702. PubMed PMID: 22492766.

9. Akintorin M. Maternal Counseling for Preterm Deliveries, Assessing an Effective Method of Counseling Bethesda, MD: National Library of Medicine; 2016 [cited 2016]. Available from: <https://clinicaltrials.gov/study/NCT02707237>.

10. Rajeev Kumar MA, Christian Castillo, Medha Kamat, LouFogg,. Randomized control study comparing different methods of counseling in mothers at risk of imminent preterm delivery Pediatric Academic Societies (PAS); May 3rd-May 6th 2014; Vancouver , Canada2014.
