# Supplementary material for: Maternal counseling for preterm deliveries, assessing an effective method of counseling: A randomized trial
Source: PLoS One. 2025 Apr 9;20(4):e0294168. doi: 10.1371/journal.pone.0294168 (PMC11981203; doi:10.1371/journal.pone.0294168)
Supplement: Attachment — Submitted filename: Rebuttal letter 3-27-2024.docx [file pone.0294168.s008.docx]

Wednesday, March 27th, 2024

To the Editor

PLOS ONE

Dear Editor,

**Ref: MATERNAL COUNSELING FOR PRETERM DELIVERIES, ASSESSING AN EFFECTIVE METHOD OF COUNSELING: A RANDOMIZED TRIAL.**

Dear Editors and Reviewers,

Thank you for your time and consideration in reviewing our manuscript. We appreciate your feedback. All comments have been addressed. Please see below for our responses.

If questions arise, please do not hesitate to email rkumar3@cookcountyhhs.org

Truly

Rajeev Kumar

Reviewer #2: Minor revisions:
1- Line 156: Wilcoxon is misspelled.

Response: The spelling has been corrected.

2- Line 156: Consider a test of the interaction effect of group by time instead of repeatedly using paired t-tests. "The paired t- test or the Wilcoxson rank signed test was used to determine if there was a statistical difference in the mean pre and post STAI tests for both intervention groups."

Response: We used professional statistician Professor Simon Galeta, who spent many years of his professional career at Iowa Department of Public Health as a statistician. He has revised the stats and instead of using Wilcoxon rank sum test, he has used the repeated measure of variance (ANOVA). A new figure has been added showing change in mean anxiety scores over time. For questions related to statistics, professor Galeta can be reached at slg@kdsol.com.

3- Line 180: The Wilcoxon signed rank test is not used to compare differences in means. It compares differences in central tendencies.

Response: The test has been removed and replaced with ANOVA.
